# Total Metastases Volume and Relative Volume Reduction After Neoadjuvant Therapy Predict Outcome After Liver Resection of Colorectal Liver Metastases

**DOI:** 10.1245/s10434-025-17295-9

**Published:** 2025-04-24

**Authors:** Florian Lindenlaub, Ulrika Asenbaum, Christoph Schwarz, Jessica Makolli, Martina Mittlböck, Stefan Stremitzer, Klaus Kaczirek

**Affiliations:** 1 Department of Biomedical Imaging and Image-guided Therapy, Medical University of Vienna, Vienna, Austria; 2 Department of General Surgery, Medical University of Vienna, Vienna, Austria; 3 Center for Medical Data Science, Institute of Clinical Biometrics, Medical University of Vienna, Vienna, Austria

**Keywords:** Colorectal cancer, Colorectal liver metastasis, Tumor burden, Metastases volume, Hepatectomy

## Abstract

**Background:**

Established clinical risk scores (CRSs) can estimate the prognosis of patients with colorectal liver metastases (CLM) after hepatic resection. However, their ability to predict outcome for patients undergoing neoadjuvant chemotherapy is limited, mainly because most included variables do not reflect a biologic response to neoadjuvant chemotherapy (NAC). This study aimed to evaluate the prognostic value of total metastases volume (TMV) and relative volume reduction (RVR) for patients with CLM undergoing perioperative chemotherapy and surgery.

**Methods:**

Liver metastases volume was semi-automatically measured on computed tomography images in 69 patients before and after NAC and compared to established CRS regarding progression-free survival (PFS) and overall survival (OS).

**Results:**

Patients with a TMV smaller than 29.5 ml before NAC and 7.5 ml after NAC showed a significantly longer PFS than those with a larger TMV (median, 31.0 vs. 13.7 months [*p* = 0.005] and 22.6 vs. 9.1 months [p = 0.013], respectively). An RVR after NAC of at least 73% was a positive predictor of PFS (median, 38.0 vs. 9.4 months; *p* = 0.004) and OS (mean, 59.5 vs. 92.5 months; *p* = 0.002).

**Conclusions:**

Total tumor volume and RVR of CLM seem to be superior to established CRS for patients undergoing neoadjuvant chemotherapy and surgery.

Colorectal cancer is one of the most common cancers worldwide,^1^ and in approximately a one fourth of the patients with a diagnosis of colorectal cancer experience liver metastases (CLM).^2,3^ Despite major advances in chemotherapeutic options, liver resection is the only potentially curative treatment.^4^

A wide range of variables determine the prognosis of patients undergoing liver resection including number and diameter of metastases, carcinoembryonic antigen (CEA) levels, synchronous metastases, and lymph node positivity of the primary tumor.^5^ Clinical risk scores (CRS) such as the Fong, Nordlinger, Nagashima scores, and recently, the tumor burden score were developed to predict outcome.^6–9^ However, most of these scores were developed before the era of perioperative chemotherapy, and their clinical applicability is limited in this setting because many of the parameters used do not represent the biologic response to neoadjuvant chemotherapy (NAC).^10^ Thus, the use of continuous variables might improve the value of CRS in the setting of perioperative chemotherapy. Additionally, the morphologic parameters used in the common CRS are only the number and diameter of metastases, with no three-dimensional variable such as the total volume of all metastases to date.^6–9^

Therefore, this study aimed to evaluate the prognostic value of total metastases volume (TMV) before and volume reduction after NAC (i.e., relative volume reduction [RVR]) and to benchmark those parameters against established CRS for patients with CLM undergoing perioperative chemotherapy and surgery with curative intent.

## Materials and Methods

This retrospective single-center study, performed at the Medical University Vienna, analyzed all patients with elective liver resection for CLM between 2005 and 2016 (*n* = 537). The study was conducted in accordance with the Declaration of Helsinki exclusion criteria, which ruled out resection of recurrent CLM (*n* = 106), resection without neoadjuvant chemotherapy (*n* = 68), lack of response to neoadjuvant chemotherapy (*n* = 28), intraoperative thermal ablation (*n* = 19), missing computed tomography (CT) or CT not suitable for volumetry (*n* = 189), and missing follow-up data (*n* = 58). After the exclusion criteria were implemented, 69 patients were eligible for analysis.

The study was reviewed and approved by the institutional review board of the Medical University of Vienna (EC no. 1110/2021).

### Perioperative Chemotherapy, Hepatic Resection, and Survival

Each patient underwent oxaliplatin- and/or irinotecan-based NAC before metastases resection. All the patients were resected with a curative intent. Patients who underwent intraoperative thermal ablation were excluded.

Progression-free survival (PFS) was calculated from date of surgery to either progression of disease or loss of follow-up evaluation. Overall survival (OS) was calculated from date of surgery to either loss of follow-up evaluation or death. Progression status was determined by means of radiologic findings according to RECIST 1.1 of intra- and extrahepatic lesions and occurrence of new intra- or extrahepatic lesions.

### Clinical Risk Scores and Other Parameters

Clinical risk scores were calculated before and after NAC. Scores were calculated only when all relevant parameters were available (Nagashima score: serosal invasion of primary tumor (≥pT3), lymph node-positive primary tumor (pN1), number of liver metastases ≥2, largest liver metastasis ≥5 cm, and resectable extrahepatic metastases; Fong score: largest liver metastasis >5 cm, disease free interval <12 months, number of liver metastases >1, lymph node-positive primary tumor, and CEA >200 ng/ml; Nordlinger score: age >60 years, serosal invasion of primary tumor (≥pT3), lymph node-positive primary tumor (pN1), disease free interval <24 months, number of liver metastases >3, largest liver metastasis >5 cm).

Based on the respective original publications, we used the original stratification of three risk groups in Nagashima and two risk groups in Fong.^6,7^ In Nordlinger, we used two risk groups due to our limitation in cohort size.^8^ Regarding the tumor burden score (TBS), we were able to calculate the TBS for every patient in our cohort and divided it into three groups: lowest 25%, 25th to 90th percentile, and highest 10%.^9^

### Multidetector CT Protocols and Volumetry

Computed tomography (CT) scans were obtained before the start of neoadjuvant chemotherapy and immediately preoperatively. The imaging protocol fulfilled the following criteria: multi-detector CT with a tube voltage adapted to the patient’s size (100–140 kVp), active tube current modulation, and intravenous injection of 50–150 ml (depending on patient’s body weight) of iodinated contrast agents (300–400 mg/ml iodine concentration).

Metastases volume was measured with syngo.CT liver analysis software (Siemens Healthineers, Forchheim, Germany) on axial images with a soft tissue kernel. The syngo.CT liver analysis software was used for manual marking, semi-automatic segmentation, and volume assessment of focal liver lesions (metastases) in either the venous or arterial phase (same phase was used before and after NAC for every patient).

### Statistical Analysis

Statistical analysis was performed using SPSS, version 28.0 (SSPS Inc., Chicago, IL, USA). Metric variables are expressed as mean or median and range.

Survival curves were computed with a Kaplan-Meier graph and compared with the log-rank test. In cases of risk scores with three ordered risk groups, the log-rank test was combined subsequently with a linear trend test. Univariate analysis using the Cox proportional hazards model then was used to quantify group differences of prognostic factors before and after neoadjuvant chemotherapy regarding PFS and OS by hazard-ratio (HR) and corresponding 95% confidence interval. A *p* value lower than 0.05 was considered to indicate statistical significance.

## Results

Patient characteristics are shown in Table 1. The study enrolled 69 patients with a median age of 62.5 years (range 33–79.1 years). The median duration of NAC was 3 months (range 1–8 months). A majority of the patients received oxaliplatin-based chemotherapy (*n* = 57, 82.6%), with 9 patients (13.0%) receiving irinotecan-based chemotherapy and 3 patients (4.3%) receiving both. Additionally, 47 patients (68.1%) received bevacizumab, 12 patients (17.4%) received cetuximab, and 8 patients (11.6%) received panitumumab. Neoadjuvant chemotherapy was part of the standard treatment, provided liver metastases were addressed in the same procedure, unless one of the following criteria was met: poor general condition, low tolerability, unfavorable overall internal medical status, patient refusal, or an acute surgical indication for the primary tumor.

**Table 1:** Patient characteristics

Median Age, years (range)	62.5 (33.0–79.1)
Sex, n (%)	
Female	27 (39.1)
Male	42 (60.9)
ASA-Score, n (%)	
1–2	49 (71.0)
3–4	20 (29.0)
Localization of primary tumour, n (%)	
Right colon	12 (17.4)
Transverse colon	1 (1.4)
Left colon	7 (10.1)
Sigmoid	19 (27.5)
Rectum	30 (43.5)
N-Stage of primary tumour, n (%)	
N0	32 (46.4)
N1	21 (30.4)
N2	16 (23.2)
T-Stage of primary tumour, n (%)	
T1	1 (1.4)
T2	13 (18.8)
T3	46 (66.7)
T4	6 (8.7)
No data	3 (4.3)
Synchronous metastases, n (%)	46 (66.7)
Median DFI	
< 12 months	48 (70.0)
< 24 months	65 (94.2)
KRAS mutation, n (%)	
Yes	16 (23.2)
No	45 (65.2)
No data	8 (11.6)
Median NAC duration, months	3 (1–8)
Adjuvant chemotherapy, n (%)	45 (65.2)
Resection Grade, n (%)	
R0	69 (100.0)
Median follow up time, months	44.4
Median PFS, months	13.8
Progression, n (%)	48 (69.6)
Median OS, months	45.6
Deceased, n (%)	27 (39.1)

*ASA* American Society of Anesthesiologists, *NAC* Neoadjuvant chempotherapy, *PFS* Progression free survival, *OS* Overall Survival, *KRAS* Kirsten rat sarcoma virus, *DFI* Disease free interval

For all the patients, an R0 resection was achieved. Adjuvant chemotherapy was received by 45 of the patients (65.2%). The exclusion criteria for adjuvant chemotherapy ruled out poor general condition, poor tolerability, poor overall internal medical status, and patient preference. The median PFS was 13.8 months, and the median OS was 45.6 months after the date of surgery.

### Tumor Volume Significantly Predicts Outcome After LR

The median tumor volume in our patient cohort was 29.7 ml before and 7.5 ml after NAC. For survival analysis, the patients were divided into two subgroups before and after NAC: group 1 (TMV lower than the median) and group 2 (TMV greater than or equal to the median). A Kaplan-Meier survival analysis showed a significant difference in PFS between the groups regardless whether volumetry was performed before (median PFS: 31.0 vs. 13.7 months; *p* = 0.005) or after (median PFS: 22.6 vs. 9.1 months; *p* = 0.013) neoadjuvant chemotherapy. No difference in OS was observed (TMV before NAC: median OS, 97.9 vs. 83.6 months [*p* = 0.713] vs. TMV after NAC: median OS, 98.0 vs. 56.6 months [*p* = 0.112]) (Table 2).

**Table 2:** Morphological data of metastases

	Before NAC	After NAC
Median number of metastases, n	3 (1–40)	2 (1–19)
Median diameter of largest metasasis, cm	4.12 (0.92–17.07)	2.56 (0–9.64)
Median volume of largest metasasis, ml	18.28 (0.39–1041.91)	4.43 (0–239.37)
Median TMV, ml	29.7 (0.4–1360.11)	7.50 (0.04–342.6)
Median RVR, %	73 (0.3–95)	

*NAC* Neoadjuvant chemotherapy, *TMV* Total metastases volume, *RVR* Relative volume reduction

**Fig. 1:**
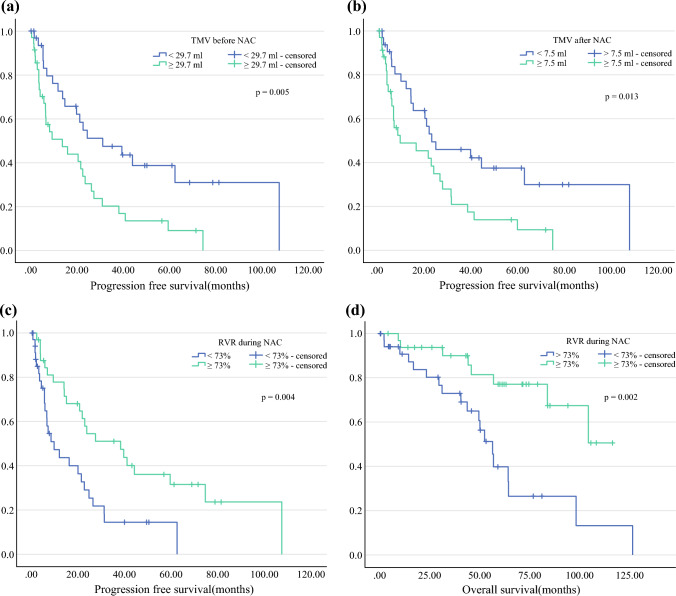
Kaplan Meier survival analysis of progression free survival (PFS) stratified by total metastases volume before (**a**) and after (**b**) neoadjuvant chemotheraphy (NAC). Patients were divided into two groups according to the median total tumor volume (TMV). Median PFS differed significantly between the two groups: 31.0 vs. 13.7 months before NAC; 22.6 vs. 9.4 months after NAC. Kaplan Meier survival analysis of PFS (**c**) and overall survival (OS) (**d**) stratified by relative volume reduction (RVR) of TMV. The patients were divided into two groups according to the median RVR of 73%. Median PFS and average OS differed significantly between the two groups (38.0 vs. 9.4 months 59.5 vs. 92.5 months respectively)

For analyzing the impact of relative tumor volume reduction, the patients were divided into two groups, with the cutoff at the median RVR (73%). The results showed a statistically significant difference in both PFS (median, 38.0 vs. 9.4 months; *p* = 0.004) and OS (mean, 92.5 vs. 59.5 months; *p* = 0.002) for the patients with an RVR of at least 73% of the initial volume (Fig. 1).

Univariate Cox analysis using the Cox proportional hazards model showed that TMV before NAC (odds ratio [OR] 1.606; 95% confidence interval [CI] 1.090–2.368; *p* = 0.017) and after NAC (OR 1.888; 95% CI 1.268–2.810; *p* = 0.002) was significantly associated with PFS, whereas no impact on OS was observed (TMV before NAC: OR 1.103; 95% CI 0.692–1.757 [*p* = 0.680] vs. TMV after NAC: OR 1.314; 95% CI 0.820–2.106; *p* = 0.257). Relative volume reduction was significantly associated with both PFS (OR 0.257; 95% CI 0.076–0.867; *p* = 0.028) and OS (OR 0.144; 95% CI 0.030–0.683; *p* = 0.015) (Tables 3 and 4)*.*

**Table 3:** Univariate Cox Regression for PFS

	*p*-value	Odds ratio	95% Confidence interval	
			Lower bound	Upper bound
TMV				
Before NAC	**0.017**	1.606	1.090	2.368
After NAC	**0.002**	1.888	1.268	2.810
RVR	**0.028**	0.257	0.076	0.867
Tumor Burden Score				1.166
Before NAC	**0.009**	1.092	1.022	
After NAC	**0.004**	1.115	1.036	1.199
Fong Score				
Before NAC	0.459	1.101	0.854	1.420
After NAC	0.654	1.072	0.790	1.455
Nordlinger Score				
Before NAC	0.858	1.022	0.801	1.305
After NAC	0.898	0.984	0.768	1.261
Nagashima Score				
Before NAC	0.268	1.153	0.897	1.482
After NAC	0.456	1.103	0.852	1.429
CEA				
Before NAC	0.859	1.000	1.000	1.000
After NAC	0.278	1.015	0.988	1.044
Number of Metastasis				
Before NAC	0.319	1.022	0.979	1.067
After NAC	**0.029**	1.082	1.008	1.161
Max. Diameter				
Before NAC	0.090	1.007	0.999	1.016
After NAC	**0.014**	1.016	1.003	1.029
Largest LiverMetastasis				
Before NAC	0.232	1.001	0.999	1.003
After NAC	0.189	1.004	0.998	1.009

Bold values are the significant results according to a two-sided *p* value of < 0.05

*NAC* Neoadjuvant chempotherapy, *PFS* Progression free survival, *CEA* Carcinoembryonic antigene, *TMV* Total metastases volume, *RVR* Relative volume reduction

**Table 4:** Univariate Cox Regression for OS

	*p*-value	Odds ratio	95% Confidence interval	
			Lower bound	Upper bound
TMV				
Before NAC	0.257	1.314	0.820	2.106
After NAC	0.680	1.103	0.692	1.757
RVR	**0.015**	0.144	0.030	0.683
Tumor Burden Score				
Before NAC	0.935	1.003	0.933	1.078
After NAC	0.820	1.012	0.914	1.120
Fong Score				
Before NAC	0.478	1.134	0.801	1.604
After NAC	0.221	1.306	0.852	2.002
Nordlinger Score				
Before NAC	0.277	1.196	0.866	1.652
After NAC	0.313	1.182	0.854	1.636
Nagashima Score				
Before NAC	0.170	1.283	0.899	1.832
After NAC	0.229	1.244	0.871	1.776
CEA				
Before NAC	0.428	0.999	0.995	1.002
After NAC	0.321	0.965	0.898	1.036
Number of Metastasis				
Before NAC	0.483	1.024	0.958	1.095
After NAC	0.800	1.013	0.917	1.118
Max. Diameter				
Before NAC	0.407	0.995	0.982	1.007
After NAC	0.891	0.999	0.982	1.016
Largest LiverMetastasis				
Before NAC	0.234	0.997	0.993	1.002
After NAC	0.461	0.997	0.987	1.006

Bold values are the significant results according to a two-sided *p* value of < 0.05

*NAC* Neoadjuvant chempotherapy, *OS* Overall survival, *CEA* Carcinoembryonic antigene, *TMV* Total metastases volume, *RVR* Relative volume reduction

### Clinical Risk Scores

We were able to calculate the Fong score for 54 patients before and 53 patients after NAC. For the Nordlinger and Nagashima scores, we were able to retrieve the relevant data for 66 patients before and after NAC. The distribution of the patients in our cohort across the different clinical risk score (CRS) categories varied, with a notable concentration at certain levels of risk. According to the Fong score, before NAC, 28 patients (51.9%) were classified as low risk (0–2 points) and 26 patients (48.1%) as high risk (3–5 points), whereas after NAC, 30 patients (56.6%) were in the low-risk and 23 patients (43.4%) in the high-risk category.

The Nordlinger score showed the patients distributed between low risk (28 patients, 42.4%) and intermediate risk (38 patients, 57.6%) before NAC, with no patients classified as high risk (5–6 points). This distribution remained similar after NAC (29 low-risk [43.9%] and 37 intermediate-risk [56.1%] patients, and still no high-risk patients).

The Nagashima score showed a wider spread, with 16 patients (29.6%) in the low-risk group (0–1 points), 38 patients (55.9%) in the intermediate-risk group (2–3 points), and 12 patients (22.2%) in the high-risk group (≥4 points) before NAC. After NAC, this distribution slightly shifted to 17 low-risk patients (30.4%), 39 intermediate-risk patients (59.1%), and 10 high-risk patients (18.5%). The limited number of patients in the highest CRS categories, particularly in the Nordlinger and Nagashima scores, should be considered when the reported *p* values in Table 4 are interpreted.

In the Kaplan-Meier analysis, none of the original CRSs were able to show significant differences in PFS or OS between the risk groups, whereas higher TBS was significantly associated with both shorter PFS and OS before NAC. After NAC, no significant impact of TBS on OS or PFS was observed in the Kaplan-Meier analysis (Table 5).

**Table 5:** Clinical Risk Scores and PFS

	Before NAC				After NAC			
	Low	Intermediate	High	*p* value	Low	Intermediate	High	*p* value
PFS (months), median								
Fong Score	38.0 (28)		22.4 (26)	0.288	38.0 (30)		22.6 (24)	0.477
Nordlinger Score	21.1 (22)	22.6 (40)	8.0 (4)	0.882	21.1 (26)	22.4 (36)	27.3 (4)	0.857
Nagashima Score	21.1 (16)	22.4 (38)	15.9 (12)	0.765	20.4 (17)	22.4 (39)	26.1 (10)	0.765
Tumor Burden Score	43.9 (17)	36.0 (46)	16.0 (6)	**0.002**	24.4 (17)	21.1 (46)	1.4 (6)	0.058
OS (months), median								
Fong Score	97.7 (28)		64.1 (26)	0.304	98.0 (30)		63.9 (23)	0.179
Nordlinger Score	83.6 (22)	56.6 (40)		0.520				0.415
Nagashima Score	98.0 (16)	63.9 (38)		0.391	98.0 (17)	56.6 (39)		0.357
Tumor Burden Score	98.0 (17)	56.6 (46)	126.2 (6)	**0.027**	98.0 (17)	64.1 (46)	126.2 (6)	0.242

Bold values are the significant results according to a two-sided *p* value of < 0.05

Fong: low 0–2pts, high 3–5pts; Nordlinger low 0–2 pts, intermediate 3–4 pts, high 5–6 pts; Nagashima low 0–1 pts, intermediate 2–3 pts, high 4 or more pts; TBS low 0–3,60 pts, intermediate 3,61 to 13.101 pts and high 13.102 or more pts

*NAC* Neoadjuvant chemotherapy, *OS* Overall survival, *PFS* Progression free survival

In the univariate Cox analysis, TBS had a statistically significant impact on PFS both before NAC (OR 1.092; 95% CI 1.022–1.166; *p* = 0.009) and after (OR 1.115; 95% CI 1.036–1.199; *p* = 0.004) NAC. No significant impact of TBS on OS was observed in the Cox analysis (Table 3).

## Discussion

Prognostic factors regarding outcome in colorectal liver metastases have been extensively researched in the past. Among the distinct clinical, biochemical, and pathologic factors currently used in prognosis scores, morphologic criteria are limited to tumor diameter and number of metastases.^6–9,11^

The prior clinical risk scores referenced in this study were published by Fong in 1996, by Nordlinger in 1999, and by Nagashima in 2006, and primarily assessed survival outcomes after surgical resection alone in an era during which chemotherapy was largely ineffective. Consequently, these scores do not reflect current therapeutic standards and are difficult to apply to the current patient population.

The radiologic techniques and tumor volume estimation used in this report were not available when those prior clinical risk scores were developed. The clinical risk scores in this analysis fundamentally differ from the prior clinical risk scores that predicted the benefit of surgery alone. A key distinction of our study is that it investigated a neoadjuvant setting, whereas the previously established scores were developed in an era before the implementation of neoadjuvant chemotherapy. The use of a two-dimensional variable such as metastases diameter can potentially lead to underestimation of the patient’s actual tumor burden.^12,13^

Recently a tumor burden score (TBS), calculated by maximum size and total number of metastases, was shown to improve discriminatory prognostic power.^9^ However, metastases of differing volumes but the same diameter are treated as equals. Metastases are rarely homogeneous in structure and often show an irregular configuration. Furthermore, in the established clinical prognosis scores and the TBS, only the largest diameter is taken into account, neglecting the size of smaller metastases.^6–8^ To overcome these issues, we investigated whether implementation of the three-dimensional variable, TMV, might further improve the prediction of outcome, especially in the neoadjuvant setting.

In our analysis, TMV was a significant prognostic factor for PFS independent of analysis before or after NAC. Higher RVR of metastases after NAC correlated with improved outcome (PFS and OS) after liver resection.

To provide a comparison with already-existing prognostic scores, we calculated Fong, Nagashima, and Nordlinger scores before and after NAC and found that none showed a significant impact on PFS or OS in the Kaplan-Meier survival analysis of the respective risk groups or the COX regression analysis. The lack of metric parameters leads to overlooking small changes that remain within threshold limits. Especially in the era of NAC, continuous morphologic criteria aid in the assessment of actual tumor burden and add significant prognostic value, as shown in the recently implemented TBS.

In our cohort, TBS proved to be a good predictor of PFS. However, in TBS, only the size of the largest metastasis is included in the calculation, allowing for another loss of information. Therefore, TMV seems to be of superior value because it provides a three-dimensional quantification of tumor burden without any loss of morphologic information. Furthermore, calculation of RVR provides a precise representation of treatment response, allowing better clinical decision-making in CLM management. Regarding OS, only RVR was a significant prognostic factor in both the Kaplan-Meier analysis and the Cox analysis.

It is important to note that due to censoring, the median OS could not be calculated for some subgroups, necessitating the use of mean survival instead. This difference in statistical methodology explains why the comparison based on TMV after NAC (in which median values could be determined) did not reach statistical significance despite a seemingly larger absolute difference, whereas the RVR-based analysis, which relied on mean survival, showed statistical significance.

Whereas the Fong, Nagashima, and Nordlinger scores require a variety of patient data (size and number of metastases, CEA levels, T and N stages, patient age, duration of disease-free interval, extrahepatic metastasis), TBS requires only two parameters (maximum size and number of metastases). Because TMV requires no further calculations or patient data, it can be calculated using a single measurement on a CT scan, thus making it an accessible way of assessing tumor burden.

Calculation of TMV and RVR is a powerful tool overcoming the aforementioned limitations of an assessment based on diameter and number of metastases. It allows a better quantification of actual extent of the disease and improves the prognostication of survival for patients undergoing perioperative chemotherapy and hepatic resection.

The future integration of artificial intelligence (AI)-driven tools could further optimize and automate the assessment of tumor volume. With AI algorithms, large imaging datasets could be processed efficiently, reducing interobserver variability and allowing precise quantification of tumor burden in real time. Such automation could lead to a standardized and more reproducible evaluation of TMV and RVR, ultimately enhancing personalized treatment planning. Furthermore, AI-based segmentation and volume measurement tools, coupled with deep learning models, could provide clinicians with rapid, accurate, and automated assessments, minimizing manual workload and improving clinical decision-making in colorectal liver metastases management.

The main limitation of this study was its small sample size. However, this study lays the groundwork for the implementation of AI algorithms that can analyze large datasets of metastatic colorectal cancer patients who undergo NAC and resection, enabling further refinement and validation of prognostic models.

Additionally, the applicability of volume software for magnetic resonance imaging (MRI) should be considered. As a more sensitive method for detecting liver metastases, MRI could provide even more precise volume assessments. In this cohort, CT was more commonly used, especially among older patients. However, the principles of volume measurement are transferable to MRI, and future studies could explore whether MRI-based TMV measurements yield even greater prognostic accuracy.

## Conclusion

Among patients with CLM, total volume and relative tumor volume reduction are valuable prognostic factors for those undergoing LR after NAC and seem superior to conventional clinical risk scores. Future prospective studies are needed to reproduce these results and link them to current prognostic scores to create an adapted scoring system including TMV or RVR.
